# Supplementary material for: Informative Regions In Viral Genomes
Source: Viruses. 2021 Jun 18;13(6):1164. doi: 10.3390/v13061164 (PMC8234400; doi:10.3390/v13061164)
Supplement: Supplementary file 1 [file viruses-13-01164-s001.zip › Supplementary Data/SupplementaryFigures.pdf]

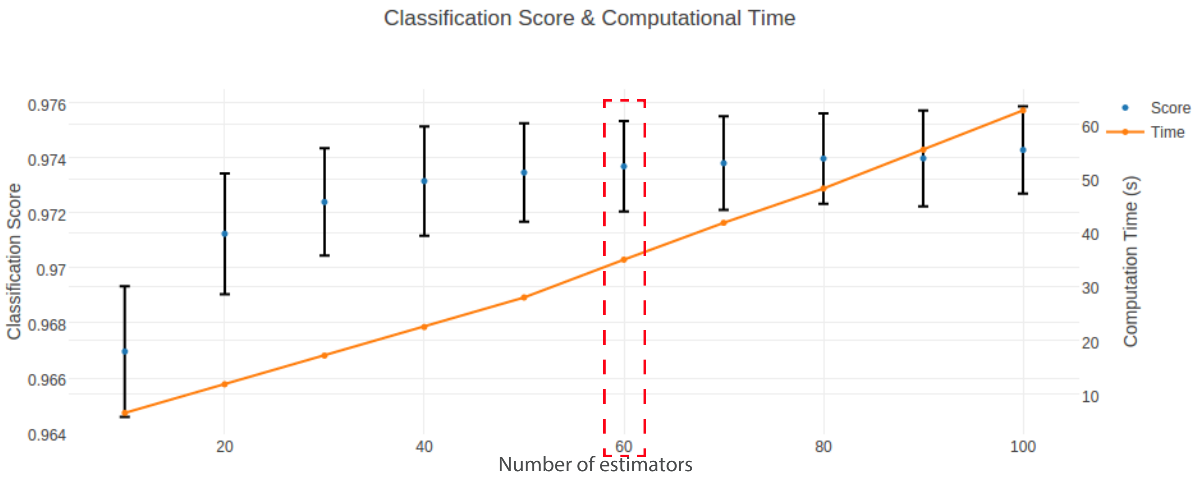

Figure S1. Model exploration. Scatter plot showing how the number of estimators/trees of the random forest (x-axis) affects the classification score (blue dots, left-y-axis) and computational time (orange dots, right y-axis). Whiskers represent standard deviation. The red dashed rectangle highlights the number of estimators used for the final model.

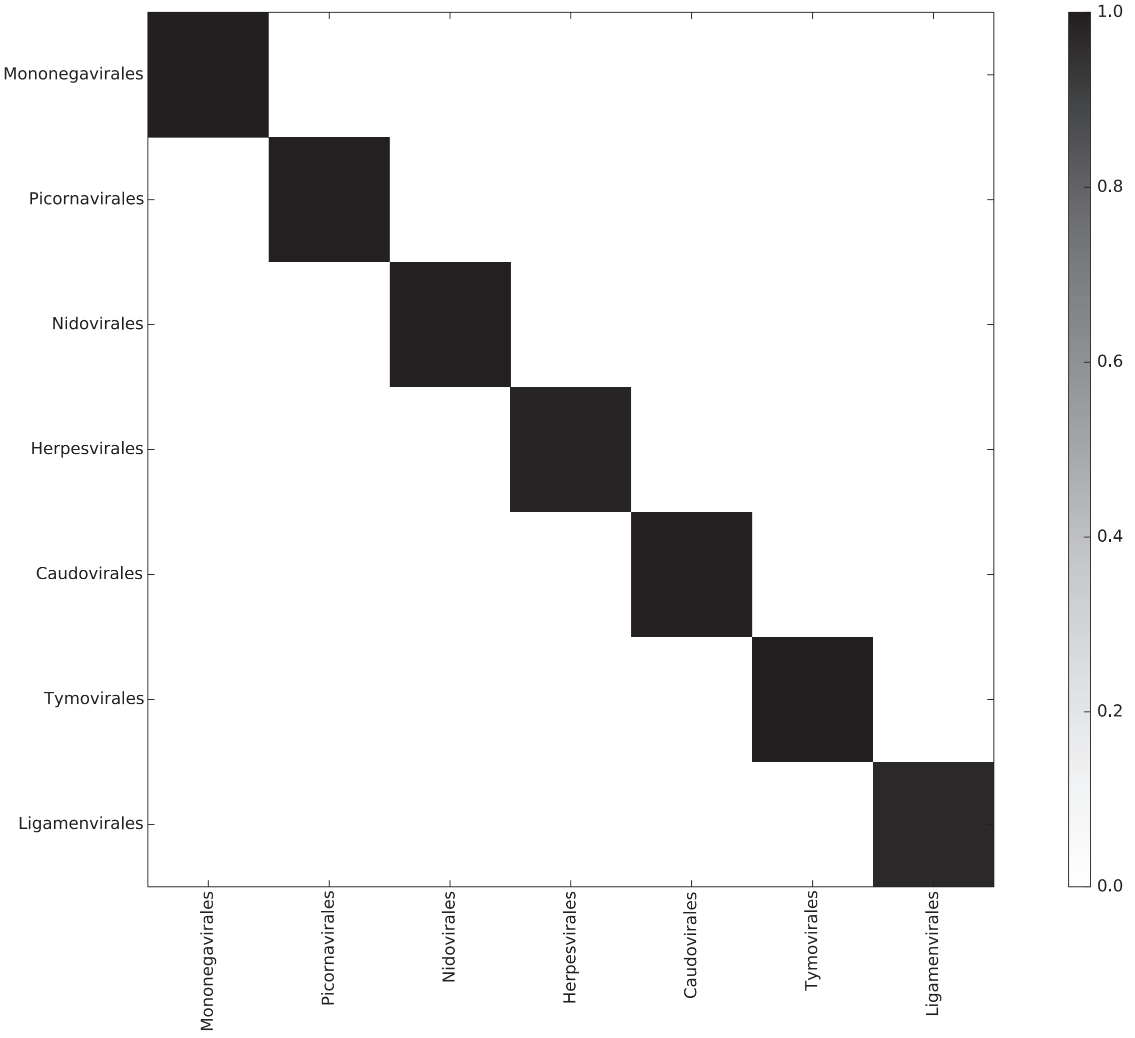

Figure S2 Random Forest accurately classifies genomes into their respective Orders. Heatmap representing the confusion matrix obtained after classifying viral genomes at the Order level. The color code indicates the proportion of genomes of the Order in the x-axis classified as a genome of the Order in the y-axis.

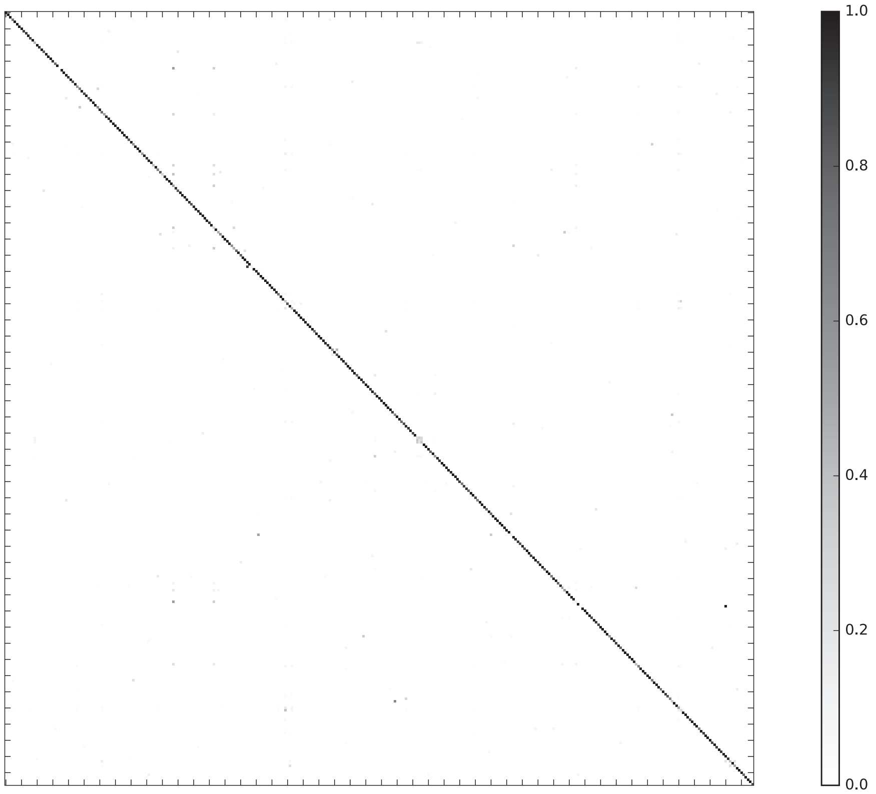

Figure S3. Random Forest accurately classifies genomes into their respective Genera. Heatmap representing the confusion matrix obtained after classifying viral genomes at the Genus level. The color code indicates the proportion of genomes of the genus in the x-axis classified as a genome of the genus in the y-axis. Genome names were removed for ease of interpretation.

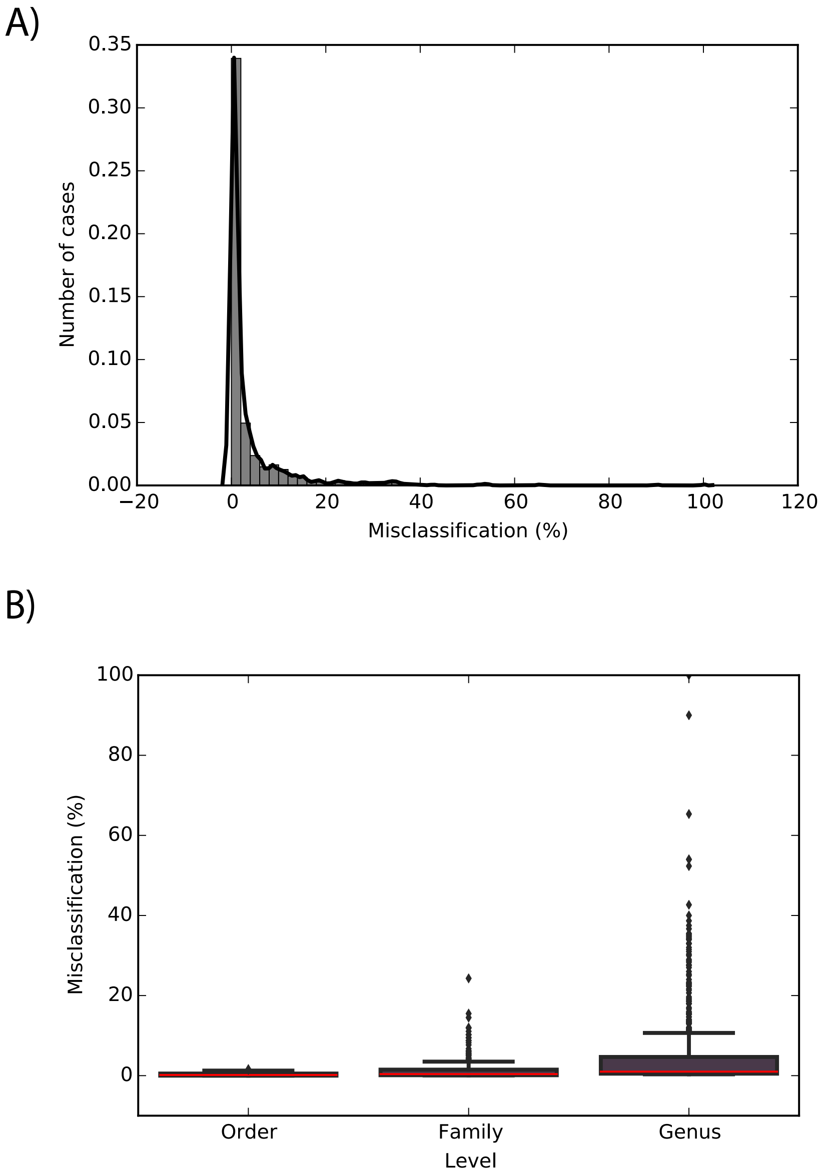

Figure S4. High misclassification is rare. (A) Distribution of the misclassification cases combined for all taxonomic levels. (B) Box plots representing the distribution of the misclassification cases per taxonomic level. Whiskers indicate 1.5 x IQR (Interquartile range), median is indicated in red.

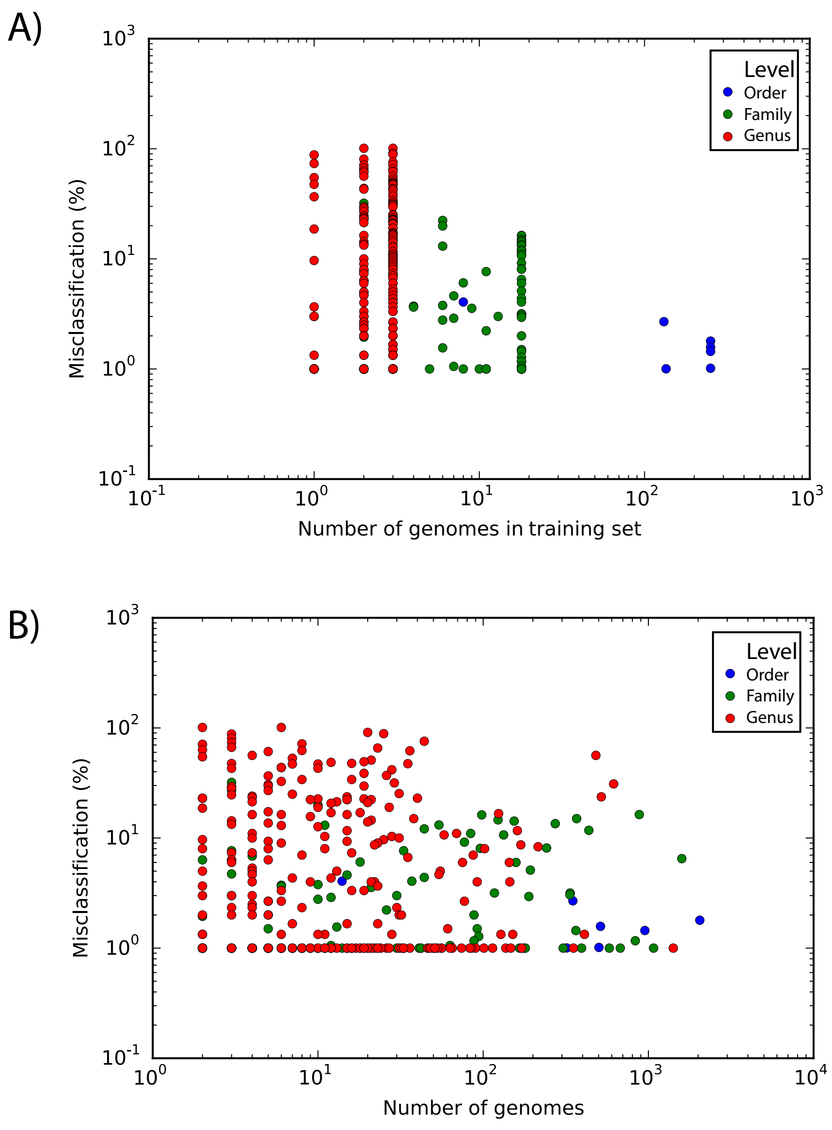

Figure S5. Misclassification in terms of the number of genomes available. Scatter plot showing (A) the number of genomes in a training iteration or (B) total number of genomes available for each taxonomic level versus the misclassification percentage. Each dot represents a taxonomical entry and the color represents the correspondent taxonomic level.

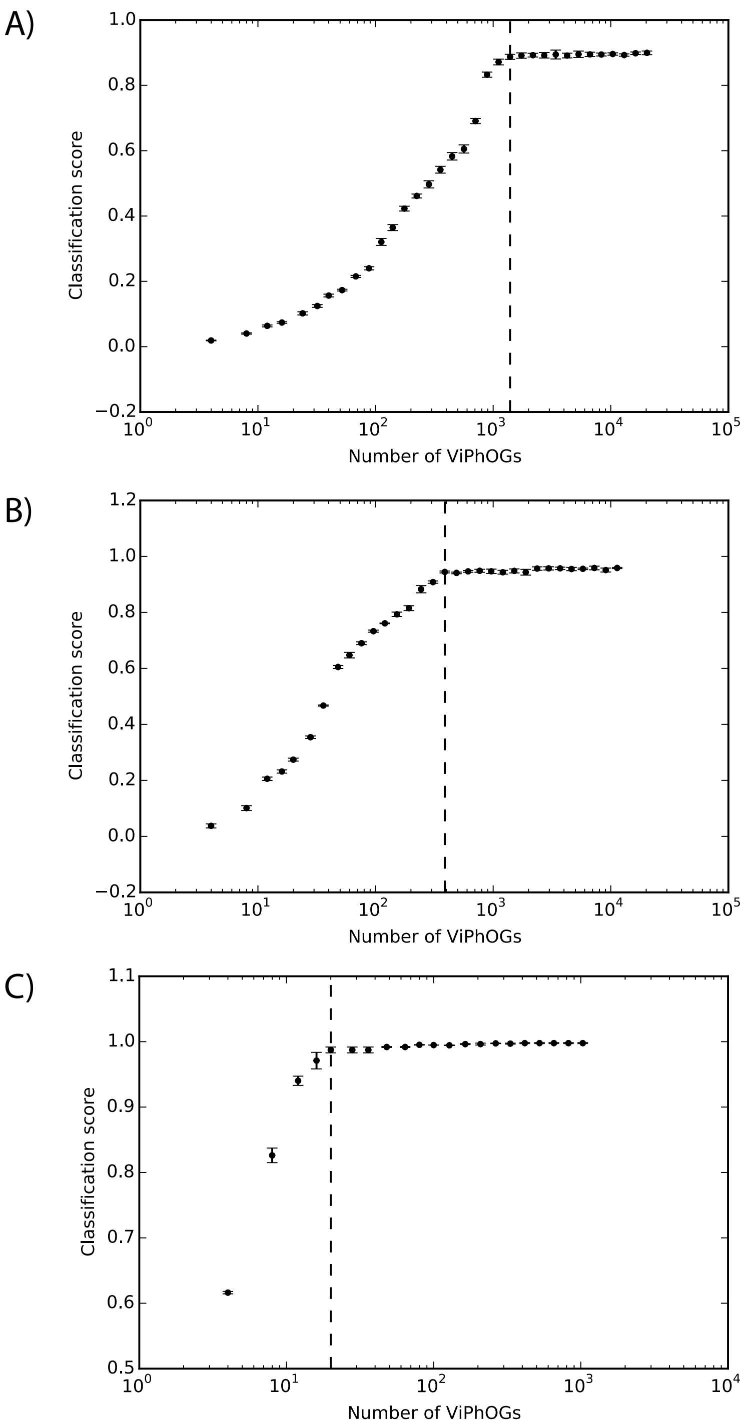

Figure S6. Selection of the informative ViPhOGs. Scatter plots showing number of importance ranked ViPhOGs versus the classification score for each taxonomic level: (A) Genus, (B) Family, (C) Order. Dashed lines indicate the number of ranked ViPhOGs chosen as informative ViPhOGs. That is, the minimum number of ranked ViPhOGs that get the highest classification score. Whiskers represent standard deviation.
